# Institutions for health care price setting and regulation: A comparative review of eight settings

**DOI:** 10.1002/hpm.2954

**Published:** 2019-11-17

**Authors:** Sarah L. Barber, Luca Lorenzoni, Paul Ong

**Affiliations:** ^1^ World Health Organization, Center for Health Development Kobe Japan; ^2^ Organisation for Economic Development and Co‐operation Paris France

**Keywords:** delivery of health care, economics, government, policy

## Abstract

**Background:**

Price setting and regulation serve as instruments to control volumes of services, while providing incentives for quality, coverage, and efficiency. In recognition of its complexity, many countries have established specific entities to carry out price setting and regulation.

**Methods:**

The aim of the study is to investigate institutions established for health care price setting and regulation and determine how countries have implemented pricing strategies. Eight settings were selected for case studies: Australia, England, France, Germany, Japan, Republic of Korea, Thailand, and Maryland in the United States. Each identified the agency responsible, their role and function, and resources for implementation.

**Results:**

In England, Japan, Korea, and Thailand, government entities conduct price setting and regulation. In Australia, France, Germany, and Maryland, independent entities were established. Their responsibilities include costing health services, establishing prices, negotiating with stakeholders, and publishing price and quality data for consumers.

**Conclusions:**

Dedicated institutions have been established to carry out costing, price setting, and negotiation, and providing consumer information. Characteristics of successful systems include formal systems of communication with stakeholders, freedom from conflicts of interest, and the mandate to provide public information. Substantial investments in price regulatory systems have been made to attain coverage, quality, and efficiency.

## 1 INTRODUCTION

Controlling the growth of health care spending while maintaining or increasing access is a major policy priority. Generally, health care spending increases at rates higher than general inflation. This is a function of both volumes of care and prices. In the United States (US), high prices alone are estimated to account for half or more of the growth in health care spending.^1^ Wide price variation can be seen both across countries and within the same country across regions and facilities.^2^, ^3^ Increases in both prices and volumes can be attributed to the adoption of new technologies, increases in income, and changes in insurance design and demographics. The demand for health services is expected to increase with population ageing.^4^ In this context, price setting and regulation serves as an instrument to control volumes of services and spending, maintain quality, and promote sustainability and efficiency.^5^, ^6^

In recognition of its complexity and importance, many countries have established or designated specific entities to carry out price setting and regulation. In this study, we investigate systems and institutions in eight settings, to understand their roles, function, and the level of resources invested to carry out price setting and regulatory tasks.

## 2 METHODS

This study focused on eight settings (Australia, England, France, Germany, Japan, Republic of Korea [Korea], Thailand, and Maryland in the US). The eight settings included in the study represent middle‐income and high‐income settings and variations in the main source of health care coverage. These settings were also chosen to represent key features of health systems that vary considerably, including the strength of regulatory systems, professional associations, and health market concentration.

For each setting, information was collected about the technical agencies responsible for price setting and regulation, their role and function, and resources required for operations and implementation. A comparison of this information is presented, followed by an analysis relevant to countries considering investing in price regulatory systems for health services. Currency conversions were done using US$ exchange rates from the World Bank database on official exchange rates for the corresponding year.^7^

## 3 RESULTS

**Figure 1:**
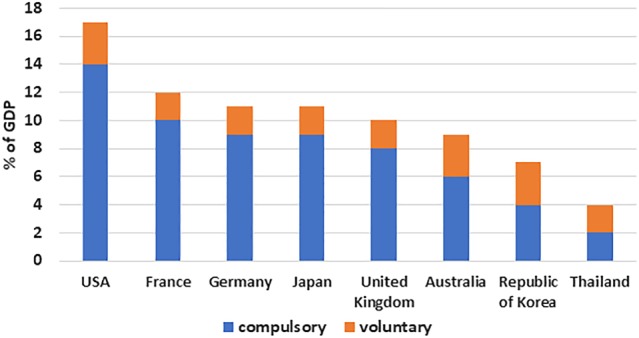
Current health expenditures as a share of Gross Domestic Product (GDP), 2016 or most recent year Source: 9. Note: Compulsory or mandatory refers to the mode of participation, whereby coverage of the population is automatic or universal, and participation is mandatory by law including social health insurance or compulsory private health insurance. Voluntary refers to coverage obtained at the discretion of individuals or firms, including voluntary private health insurance. Spending on capital items is not included [Colour figure can be viewed at http://wileyonlinelibrary.com]

Figure 1 illustrates the level of health spending for each of the eight settings in the study. The total amount of resources for health varies widely across these settings. Current health expenditure as a share of GDP ranges from 17% in the USA to less than 4% Thailand (Figure 1). The source of most spending in all settings is compulsory (ie, set aside by the government for certain health programs or initiatives).

Australia, England, and Thailand's Universal Coverage Scheme have systems of health coverage based on residence or citizenship. The other settings have employment‐based contributory health coverage and vary by the number of payers. In Korea, there is a single payer system, whereas in France and Japan, multiple payers exist with automatic (compulsory) affiliation. In Germany and the US, multiple payers exist with choice of affiliation (Table 1).

**Table 1:** Main source of health care coverage for case study settings

Main Source of Basic Health Care Coverage		Country
**Citizen entitlement**		Australia, Thailand (UCS, CSMBS), England
**Employment‐based coverage**	Single payer	Republic of Korea, Thailand (SHI)
	Multiple payers with automatic affiliation	France, Japan
	Multiple payers with choice	Germany, USA

Sources: 10, 11. Abbreviations: CSMBS, Civil Servant Medical Benefits Scheme; SHI, Social Health Insurance; UCS, Universal Coverage Scheme.

England, Japan, Republic of Korea, and Thailand have designated the tasks for price setting and regulation under the responsibilities of the government ministry (Table 2). In England, the National Health Service (NHS) responsibilities for price setting are shared by NHS Improvement and NHS England, who have been working under a joint operating model since April 2019. Their responsibilities are comprehensive and include commissioning health care services, contracting for health care providers, and supporting Clinical Commissioning Groups that plan and pay for local health services. They are also responsible for calculating prices from cost data collected from all NHS health service providers. Public consultation on the price‐setting methodology is formalized with internal stakeholders, as well as the external clinical community, NHS service providers, and Clinical Commissioning Groups to ensure that new proposals make clinical sense and are practical to implement. If more than 66% of commissioners or providers object, the regulated prices must be referred to the Competition and Markets Authority or a new consultation is conducted. All costing and price information is in the public domain, and an impact assessment is published alongside each national tariff. Pricing activities engage the work of an estimated 75 staff in NHS England.

**Table 2:** Technical agencies mandated for price setting, where located within the government

Setting	Institution Responsible	Tasks	Scope of Data Collection for Costing	Human Resources
**England**	National Health Service (NHS) Improvement, NHS England	NHS Improvement has broad responsibilities for commissioning health care services in England, including contracting health care providers, supporting Clinical Commissioning Groups that plan and pay for local services, as well as calculating prices.	The scope of data collection includes acute inpatient and outpatient care excluding psychiatric services, emergency care, and rehabilitation. Data are collected annually from all 232 National Health Service providers in England (80 NHS trusts and 152 NHS foundation trusts).	NHS England and NHS Improvement employs approximately 7500 staff, and some 75 staff work in the two pricing teams.
**Japan**	Ministry of Health, Labour and Welfare (MoHLW), under the Bureau of Medical Affairs	The Bureau of Health Insurance serves as a secretariat to the process and that the item revisions are equal to the global budget. It collects data on revenue and expenditures to inform this process. It also conducts negotiations with the medical professional associations and hospital groups.	The scope of data collection includes inpatient and outpatient services, pharmaceutical, and medical devices. Revenues and expenditures are collected every 2 years for health services and annually for pharmaceuticals from the Health Economic Survey of facilities. Volume is collected from the National Claims Database.	Staff in the Medical Affairs Division number 84 in total, including 20 physicians, 2 dentists, 2 pharmacists, 2 nurses, and 12 career bureaucrats, with the rest being administrative staff.
**Republic of Korea**	National Health Insurance Corporation (NHIS), Health Insurance Review and Assessment (HIRA), Insurance Policy Deliberation Committee (HIPDC), National Health Insurance Service (HIRA), Ministry of Health	The HIRA costs and analyzes provider behavior related to pricing. It manages the Healthcare Review and Assessment Committee, responsible for reviewing benefits design. The HIRA and each provider association negotiate fees. The HIPDC approves major decisions about health insurance, including pricing.	The scope of data collection includes inpatient and outpatient services. Participating providers provide data on an annual basis.	The NHIS has about 14 000 workers. HIRA has about 2500 staff, one headquarters (22 departments), one research institute, and seven regional offices. The Health Care Review and Assessment Committee consists of approximately 1050 members, with a maximum 50 full‐time members. HIRA also has various expert committees to support technical decisions.
**Thailand**	National Health Security Office (NHSO), National Health Security Board (NHSB)	The subcommittee on financing under the NHSB analyzes the unit costs, utilization rates, high cost interventions and all other benefit packages as approved by the NHSB, and proposes a capitation budget.	The scope of data collection includes inpatient and outpatient services (eg, staffing, medicines, diagnostics, and capital depreciation costs). Some 900 public hospitals provide data on a routine basis.	NHSO has 881 staff (464 in the HQ office and 467 in 13 regional offices). Staff generate the annual budget, monitor and purchase services, and improve access and financial risk protection to its 47 million members. The total administrative cost is 1.49% of total budget (average 2003‐2019).

Source: 12.

In Japan, the Bureau of Medical Affairs (including 84 staff of the Ministry of Health, Labour, and Welfare) sets forth the biennial revision of the fee schedules and authorizes negotiations between the Japanese Medical Association and other stakeholders. The process is driven by the Prime Minister, who sets the global revision rate in the biennial revision of fees and the conditions of billing that establish the human resource requirements and patient conditions. The Bureau of Health Insurance serves as the secretariat to ensure that the cumulative effect on item revisions is made equal to the global budget. It negotiates with the Japanese Medical Associations, hospital associations, and specialist groups about the details of the revisions. Such revisions are informed by data from the health economic survey of facilities, and the national claims database. This consultation process takes place within the Central Social Medical Care Council, which is composed of seven members from payer groups (including social health insurance, business, and labor), seven members from provider groups, six members who represent public interests, and 10 specialists representing professional associations and industry. The government publishes online a report surveying patient satisfaction indicators nationally.

In the Republic of Korea, the Health Insurance Review and Assessment (HIRA) under the Ministry of Health costs and analyzes provider behavior related to pricing. One of the key institutions under HIRA is the Healthcare Review and Assessment Committee, which plays an important role in the benefits design, review, and assessment. It also negotiates fees with each health care provider association, including physicians, hospitals, and pharmacists. The Health Insurance Policy Deliberation Committee (HIPDC) approves major decisions about health insurance, including contribution rates, benefit packages, as well as pricing. Price and volume data are collected regularly to inform this process. Regulated prices and quality measures are published online.

The HIPDC consists of 25 members, chaired by the Vice Minister of Health and Welfare. Eight members represent payers (including labor unions, employer associations, civic groups, consumer associations, farmers associations, and self‐employment associations), eight from health care professional associations (representing medical doctors, hospitals, traditional medicine practitioners, dentists, pharmacists, nurses, and pharmaceutical manufacturers), and eight experts and public agency representatives (from Ministries of Health, Strategy and Finance, Health insurance, and independent experts). Some 50 full‐time staff in Korea work on benefits design and conduct negotiation with health care provider associations about fees.

In Thailand, the responsibilities for pricing for the Universal Health Coverage Scheme fall under the National Health Security Board. Its National Health Security Office (NHSO) is a state agency under the supervision of the Public Health Minister. A subcommittee on financing analyzes the unit cost, utilization rate, high cost interventions, and all other benefit packages as approved by the Board and proposes a capitation budget. The budget for the Universal Coverage Scheme is evaluated by all relevant actors including the Ministry of Finance, Bureau of Budget, technical experts, and health care provider representatives.

In the other four settings, independent agencies were established with the responsibility for developing and updating hospital prices and fee schedules. This has occurred in Australia, France, Germany, and the state of Maryland in the United States (Table 3).

**Table 3:** Technical agencies established for hospital price setting

Setting	Entity	Responsibilities	Scope of Data Collection for Costing	Resources
Australia	Independent Hospital Pricing Authority (IHPA)	The IHPA is responsible for activity‐based costing, the classification system (AR‐DRGs and for subacute and nonacute services in the Australian National Sub‐acute and Non‐Acute Patient Classification), data collection on activity (the National Hospital Data Collection), calculating costs (with a standard framework for costing activities, ie, the Australian Hospital Patient Costing Standards).	Data collection covers inpatient care, subacute, emergency, and outpatient services. All public hospitals participate every 1 to 2 years. A separate system of data collection is undertaken from 91 (out of 630) private hospitals on a voluntary basis.	For the financial year 2017/2018, the IHPA's total expenses were AUS $17.9 million (US$ 23.4 million), and 42 staff were employed.
France	Technical Agency for Hospital Information (ATIH)	The ATIH is an independent public administrative institution cofunded by the government and national health insurance funds, under the control of the Social and Finance Ministries. It collects data and categorizes diagnosis‐related groups (DRGs).	Data collection covers acute inpatient and outpatient care excluding psychiatric services, emergency care, and rehabilitation. Some 135 hospitals participate on a voluntary basis annually.	For the financial year 2017, the ATIH employed 118 staff, and its expenses amounted to EUR 29.4 million (US$ 24.9 million).
Germany	Institute for the Hospital Remuneration System (INEK)	The INEK is jointly supported by the Federal Association of Sickness Funds, the Association of Private Health Insurance, and the German Hospital Federation. It receives data from hospitals annually to develop the Case Fee Catalogue for the following year. Hospital data follow a standardized cost accounting approach to calculate the costs of treating individual patients. Participating hospitals receive a fixed allowance for sharing the cost accounting data.	Data are collected about medical treatment, nursing care, pharmaceuticals and therapeutic devices, board and accommodation, and excluding intensive and emergency care. Some 300 hospitals participate on a voluntary basis annually.	All hospitals pay a diagnosis related group (DRG) system contribution per hospital case, and the InEK receives one‐third of the total contribution to fund their activities. In 2017, the INEK's estimated budget was EUR 8.2 million (US$7.3 million). It employs approximately 50 staff.
Maryland, USA	Health Services Cost Review Commission (HSCRC)	The HSCRC establishes hospital rates to promote cost containment, access to care, equity, financial stability, and hospital accountability. All Maryland hospitals are paid based on the rates established by the HSCRC. These rates are updated each year based on multiple factors, including the Medicare “market basket” forecast, economic conditions, productivity improvements, changes in case mix, and the previous year's performance.	Data include inpatient and outpatient services among participating providers collected annually.	The HSCRC employs 39 full‐time staff, with a budget of US$14.1 million funded by fees collected from hospitals.

Source: 12.

In Australia, the Independent Hospital Pricing Agency reports to a board chosen by the national and state and territory governments. It has broad responsibilities for activity‐based costing, the classification system, data collection, and calculating costs based on the Australian Hospital Patient Costing Standards. The National Hospital Data Collection effort covers inpatient, outpatient, and emergency care among public hospitals at 1 to 2‐year intervals. A separate system of data collection is undertaken from a selection of private hospitals that volunteer to participate. Consultation and stakeholder feedback is an integral part of the price setting process. The pricing authority works with a Jurisdictional Advisory Committee and a Clinical Advisory Committee in developing its systems and analyzing data. Its pricing framework establishes various principles, including transparency, and the framework itself is reviewed annually in consultation with the federal government, states, and territories. There is also a period of public consultation, and the studies are published on the authority's website. It publishes both price and quality information for the public. In 2017/2018, it employed 42 staff, and its operating budget was AUS$ 17.9 million (US$ 23.4 million).

In France, the Technical Information Agency of Hospitalization (ATIH) was created in 2002 as an independent public administrative institution, which is cofunded by the government and the national health insurance funds. It collects data on hospital activity in order to establish a national schedule and undertakes financial analysis of health care facilities and of the health system. Some 135 hospitals participate voluntarily on an annual basis in data collection efforts that covers acute inpatient and outpatient care. In 2017, it employed 118 staff, and its budget was approximately EUR 29.4 million (S$ 24.9 million).

In Germany, the Federal Association of Sickness Funds, the Association of Private Health Insurance, and the German Hospital Federation established the Institute for the Payment system in Hospitals (InEK). It is not an independent entity, but a public entity supervised by the three parties that oversees the hospital payment system. Generally, these institutes are responsible for the technical details of price determination, including establishing common frameworks for price estimation and collecting directly or commissioning the collection of data. Notably, the InEK neither collects or commissions data; it defines the costing approach, produces guidelines, and oversees data collection from some 300 hospitals participating on a voluntary basis annually. It employs approximately 50 staff. To fund the operations of the Institute, the three parties negotiate annually an amount in which hospitals pay a DRG system contribution per case. Participating hospitals receive two‐thirds of the contribution, whereas the InEK receives one‐third. In 2017 and 2018, the contributions amounted to EUR 1.30 and EUR 1.31 per case, respectively. Given that the number of cases amounted to over 19 million in 2017, this implies that the InEK received a budget of EUR 8.2 million (US$ 7.3 million).

Maryland established the Health Services and Cost Review Commission (HSCRC) in 1976 to regulate hospital fees for all hospitals, based on a list of approved fees for specific services and departments. It works closely with the Maryland Department of Health, and its seven commissioners are appointed by the Maryland governor. The agency is thus independent, and its decisions are not reviewed by the legislative or executive branches. The Commission has an Advisory Committee and technical working groups that conduct formal expert technical consultation. The Commission is responsible for updating the rates annually and publicly disclosing hospital data. It publishes an online price guide and a hospital performance evaluation guide. It employs some 39 staff and has a budget of US$ 14.1 million funded by hospital fees.

## 4 DISCUSSION

This study found variations in how systems for health care price setting and regulation were established. In four of the eight settings in this study (England, Japan, Korea, and Thailand), the responsibilities for price regulation were established under the relevant government ministries. The benefits of this approach are the linkages among payment systems for primary and inpatient care, and the close alignment between payment systems and government goals. This is an advantage in settings where the government is a credible authority to the public.

In four other settings (Australia, France, Germany, and Maryland in the USA), entities with the legal authority to set up and control hospital payment rates were established. Broadly, the mandate of these agencies is to develop a credible price schedule for hospitals. This includes grouping and ordering services based on their complexity, taking into consideration the available health resources, burden of disease, and clinical protocols and pathways.

Characteristics of successful systems include political independence, formal systems of communication with stakeholders, credibility in the eyes of the public, freedom from conflicts of interest, and political standing to resist both industry capture and political pressures. In some cases, such entities have independent sources of funding that are separate from general revenues. This has occurred in Germany and Maryland in the USA, for example.

While situations vary, independent agencies may have more freedom from conflicts of interest, and the political standing to resist industry and regulatory capture. Moreover, the establishment of national independent agencies can help to promote comparability and harmonization of clinical classifications across hospitals. In some settings, such harmonization applies across both public and private sectors, whether through the contracting of services or price benchmarking. Finally, clearly delineating the technical task of establishing the price schedule from the political process of negotiating payments to health care providers has also been recommended.^8^ This is particularly true in the case of independent authorities.

An important issue is how to make use of all health resources available to attain coverage and efficiencies. Price setting for only one part of the health system (either public or private) could create incentives for providers to shift care to other settings that are not subject to price regulation. This would diminish the impact of pricing policies on coverage and desired outcomes. A comprehensive price setting system could be used to create a level playing field and eliminate the fragmentation across public and private sectors. In this sense, price schedules are a public good, whereby private health plans may use prices set by the government as benchmarks. Given finite resources for health, price regulation can be used to promote greater value for all payors, both public and private.

A balance must be found between maintaining dialogue with stakeholders, including the health industry, while also observing objectivity and independence. To address this challenge, formal consultation processes have been implemented in each setting that involve stakeholders in the discussion of the base price and the cost elements that it covers. Feedback from health care providers involved in care provisions may ensure acceptability of the regulated fees.

Each of the agencies also demonstrated a mandate to inform consumers about prices and quality. These agencies average or median of hospital prices for individual services, and some report total and out‐of‐pocket costs for care episodes. Publishing service prices charged by health care providers is one means to help consumers make informed choices. Price and quality information also inform active purchasers of health care and can, in some cases, control overall spending and reduce price variation for routine services.

In each setting, pricing policies have been used not only to ensure adequacy in covering the costs of delivering services but also to provide financial incentives for health care providers. This study confirms that pricing and payment systems are powerful tools to drive broader health system goals. Hence, investing in formal mechanisms enables price setting and price regulation to be used as instruments to achieve broader system goals.

## FUNDING

Funding was provided by the World Health Organization Centre for Health Development grant numbers K18013 and K18014.

## CONFLICT OF INTEREST FOR ALL AUTHORS

None declared.

## References

[1] Martin A , Lassman D , Whittle L , Catlin A . Recession contributes to slowest annual rate of increase in health spending in five decades. Health Affairs. 2014;33(1):67‐77. 21209433 10.1377/hlthaff.2010.1032

[2] Lorenzoni L , Koechlin F . International Comparisons of Health Prices and Volumes. New Findings. OECD Publishing; 2017.

[3] Cooper Z , Craig SV , Gaynor M , Reenen JV . The price ain't right? Hospitals prices and health spending on the privately insured. National Bureau of Economic Research Working Paper 21815; 2018. 10.1093/qje/qjy020 PMC7517591 32981974

[4] European Commission. The 2018 Ageing Report, Economic and Budgetary Projections for the 28 EU Member States (2016‐2070). Institutional Paper 079; 2018.

[5] Anderson GF , Reinhardt UE , Hussey PS , Petrosyan V . It's the prices, stupid: why the United States is so different from other countries. Health Affairs. 2003;22(3):89‐105. 12757275 10.1377/hlthaff.22.3.89

[6] Anderson GF , Hussey P , Petrosyan . It's still the prices, stupid: why the US spends so much on health care, and a tribute to Uwe Reinhardt. Health Affairs. 2019;38(1):87‐95. 30615520 10.1377/hlthaff.2018.05144

[7] World Bank . 2019 Official exchange rate (Accessed 1 August, 2019 https://data.worldbank.org/indicator/PA.NUS.FCRF?most_recent_year_desc=true )

[8] Kumar A , de Lagasnerie G , Maiorano F , Forti A . Pricing and competition in Specialist Medical Services: an overview for South Africa. Organisation for Economic Cooperation and Development Working Papers, 2014 (70), OECD Publishing.

[9] World Health Organization (WHO) . 2019 Global Health Expenditure Database (http://apps.who.int/nha/database/Select/Indicators/en , Accessed 13 April 2019).

[10] Paris V , Devaux M , Wei L . Health systems institutional characteristics: a survey of 29 OECD countries. OECD, Directorate for Employment, Labour and Social Affairs, OECD Health Working Papers 50; 2010.

[11] Jongudomsuk P , Srithamrongsawat S , Patcharanarumol W , Limwattananon S , Pannarunothai S , Vapatanavong P , et al. The Kingdom of Thailand Health System Review. 5(5). Manila: World Health Organization, Regional Office for the Western Pacific, 2015.

[12] Barber SL , Lorenzoni L , Ong P . Price setting and price regulation in health care: lessons for advancing universal health coverage. Geneva: World Health Organization, Organisation for Economic Cooperation and Development; 2019 License: CCBY‐NC‐SA 3.0 IGO.

